# Migration and political polarization in the U.S.: An analysis of the county-level migration network

**DOI:** 10.1371/journal.pone.0225405

**Published:** 2019-11-22

**Authors:** Xi Liu, Clio Andris, Bruce A. Desmarais

**Affiliations:** 1 Department of Geography, Pennsylvania State University, University Park, PA, United States of America; 2 Department of Political Science, The Institute for CyberScience, Pennsylvania State University, University Park, PA, United States of America; Georgia State University, UNITED STATES

## Abstract

**Research question:**

From gridlock in lawmaking to shortened holiday family dinners, partisan polarization pervades social and political life in the United States. We study the degree to which the dynamics of partisan polarization can be observed in patterns of county-to-county migration in the U.S. Specifically, we ask whether migration follows patterns that would lead individuals to homogeneous or heterogeneous partisan exposure, using annual county-to-county migration networks from 2002 to 2015. Adjusting for a host of factors, including geographic distance, population, and economic variables, we test the degree to which migration flows connect counties with similar political preferences.

**Findings:**

Our central finding is that over the period studied, county-to-county migration flows connect counties with similar partisan voting profiles. Moreover, partisan sorting is most pronounced among the most politically extreme counties. The implication of this finding in the context of partisanship is that U.S. migration patterns reinforce partisan sorting, limiting the degree to which individuals will experience cross-the-aisle local social contacts through spatial interaction. This finding builds on existing research that has documented (1) that individuals prefer to move to and live in locations inhabited by co-partisans, and (2) that local geographic areas have become more polarized in recent decades. Our results indicate that large scale patterns of polarized migration flows serve as a potential mechanism that contributes to geographic partisan polarization.

## Introduction

The national political environment in the United States has grown increasingly polarized between Democrats and Republicans in recent years. From the behavior of legislators in the United States Congress [1–3] to individual consumption habits [4], and even the length of family holiday dinners [5], political party affiliation shapes a wide variety of behavioral phenomena. The Democrat / Republican divide does not just represent a national divergence, but has grown to shape local politics as well [6], suggesting that pockets of divides, and the flows between them, may be a useful way of viewing political polarization. In the current study, we investigate the degree to which geographic migration patterns follow other divides between Democrats and Republicans.

The way in which migration and partisanship are related plays a significant role in shaping the national political landscape in the United States. The national legislature, the presidency, and sub-national elected offices, are allocated according to rules that reflect the geographic distribution of partisan support [7]. For example, representation in the Senate (entirely) and the Electoral College (in part) are allocated evenly across the states. If politically extreme areas on one side of the partisan aisle cluster into small states, that side of the aisle will be disproportionately represented (relative to the distribution of voters) in the national political landscape [8]. To understand the factors shaping party control in the United States, it is critical that we understand the relationship between internal migration and partisanship.

There is a substantial literature that finds that partisanship is both correlated with and causes decisions that are closely related to migration. Research to date finds that individuals exhibit a general tendency towards partisan sorting when deciding where to move. Individuals are more likely to move to congressional districts that match their partisan affiliations [9, 10]. Individuals value residential real estate more highly when they learn that a property is in a neighborhood populated by co-partisans [11], and are generally more favorable towards areas in which they are in the partisan majority [12]. In a study of a region of six U.S. states, Carlson and Gimpel (2019) [13] find that substantial minorities of migrants, including many who change their official political party affiliations, migrate in ways that are consistent with intentional partisan sorting. In a result that is closely related to our analysis, Charyyev and Gunes (2019) [14] find that county-level migration flows in the U.S. point more heavily to Republican and politically moderate counties. The literature also indicates that counties and other geographic areas are becoming more polarized along party voting lines—a dynamic that is related to county in- and out-migration rates [15, 16]. In summary, the existing research has demonstrated a general tendency for individuals to prefer living among co-partisans, and that local areas have grown more partisan in recent years.

These trends raise a question of whether the increasing polarization of local areas is driven by polarized migration flows. To understand whether partisan migration flows drive geographic polarization, we need to test for large-scale patterns of sorting via migration flows. We fill this gap in the current research by studying recent historical patterns in county-to-county migration flows. We present an analysis of the association between county-level presidential party voting results and county-to-county annual migration flows from 2002 to 2015. In this analysis, we are careful to adjust for the effects of other factors, such as economic conditions, that may drive migration flows to attractive places. Migration in year *i* refers to address changes reported to the Internal Revenue Service (IRS) between year *i* + 1 and year *i*. This period captures two different presidential administrations (George W. Bush and Barack Obama), several changes in party control of Congress, and one redistricting period (2010). It is also influenced by the 2016 election of Donald Trump.

We first visualize migration flows plotted against the partisan vote breakdown in origin and destination counties. We find a consistent and striking pattern in this analysis—that polarized migration flow is common, and that it is strongest among counties at the political extremes. We then use a regression approach to test whether this pattern is consistent after adjustment for other social and economic factors affecting migration, and more precisely, estimate the form of the relationship between partisanship and migration. We find a consistent pattern, wherein migration flows are polarized and the most intense polarization is driven by extremely partisan counties.

We conceptualize the study of county-to-county migration as a network analysis problem. In doing so, we follow a growing body of work that considers migration from a network science perspective [17–19]. We draw upon the network perspective to make use of two concepts. The first is that of “homophily”, or the tendency for units that are alike to interact at greater volumes than those that are dissimilar [20, 21]. The second concept upon which we draw is “complex dependence”, a perspective that acknowledges the tendency for relationships (e.g., migration ties) to be dependent upon each other (e.g., one person moving to a county may induce others to move to the same county) [22, 23]. Both concepts inform the analysis that we present below.

## Materials and methods

As reviewed above, the literature offers substantial evidence that (1) information about the political environment of a local area affects individuals’ assessments of suitability and satisfaction, and (2) in several regions and areas, migration patterns fit with models of geographic political sorting. Our objectives are to build upon this research in two ways. First, we seek to scale our analysis to cover the contiguous 48 states, and most of the U.S. population. Second, we seek to advance our understanding of the functional form that most accurately characterizes the political components of county-to-county migration. We use data from several publicly available sources. We source county-to-county migration flows from the U.S. Internal Revenue Service (IRS) Statistics of Income (SOI) indicators, using number of exemptions to represent migrants. Our data span 14 years, from 2002 to 2015 and are aggregated in two-year summed increments. We use counties of population 20,000 or more in the 2010 Census for the contiguous 48 states, resulting in 1,834 counties. The 1,834 counties used in our analysis preserves 94.3% of total migration flows (weighted by migrants) and 88.1% of the total migration connections (un-weighted, i.e. unique network edges) (see S1 Appendix for more detail).

**Fig 1:**
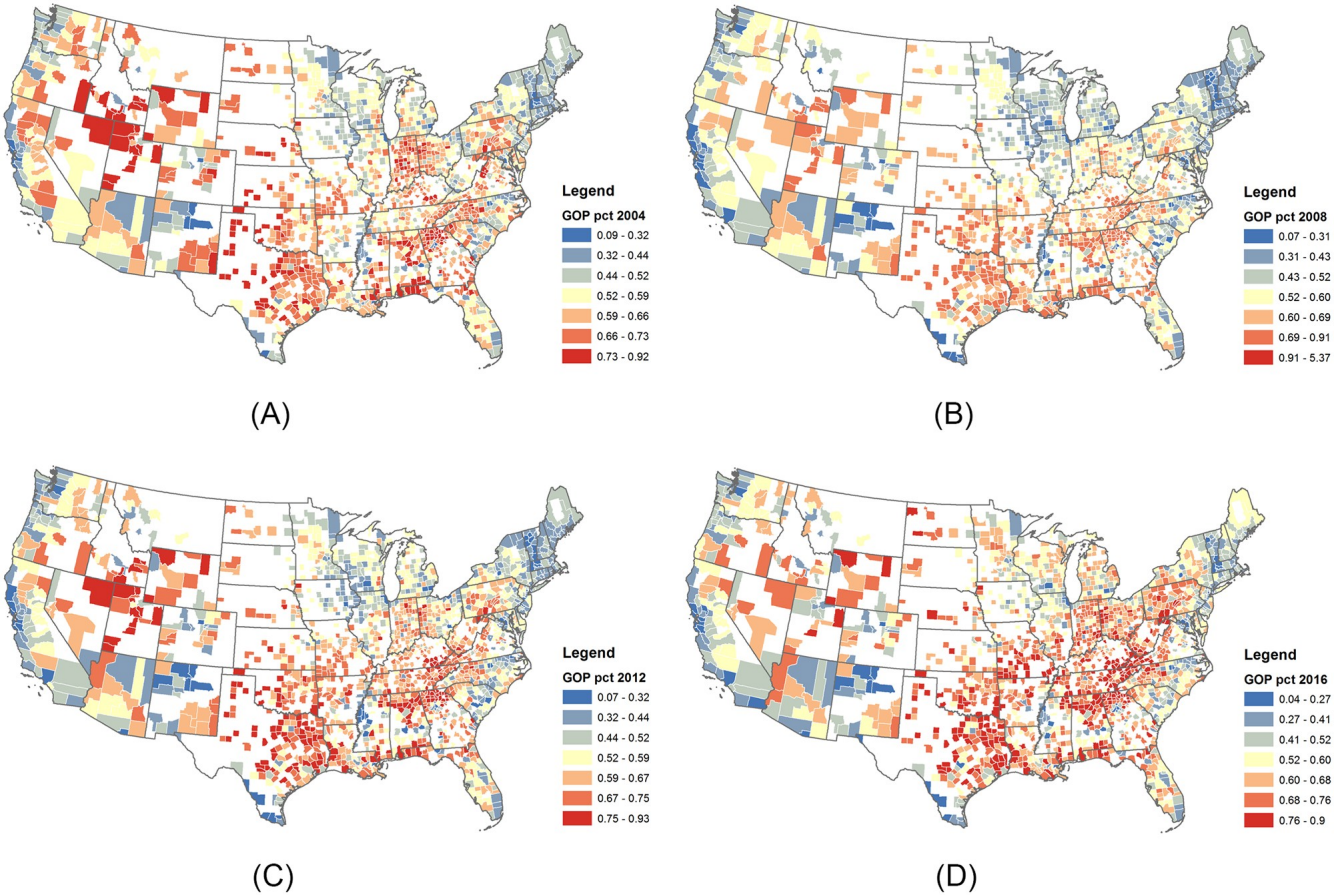
County-level vote maps. Counties with more than 20,000 residents in 2010 are used in the analysis. The percentage of votes for GOP are mapped for years 2004 (A), 2008 (B), 2012 (C), and 2016 (D), showing regional trends.

County-level presidential voting outcomes are sourced from a open dataset County Presidential Election Returns [24] for years 2004, 2008, 2012, and 2016 (Fig 1). Data on number of employees in different industries, such as agriculture, technology, service industry, education, and military professionals come from the U.S. Bureau of Labor Statistics, using the two digit North American Industry Classification (NAICS) codes of 20 major industries for years 2013 and 2016. Data on population, median home value, percent of residents with bachelor’s degrees, median household income, and unemployment rates were gathered from the U.S. Census American Community Survey (ACS) or Decennial Census for years 2000, 2009, 2012, and 2016. Information on matching between independent variable values for each 2-year migration estimate is detailed in the S1 Appendix. Regarding GIS data, county centroids were computed in ArcMap using county shapefiles from U.S. Census TIGER Line files, as was Euclidean distance between counties.

To generate heatmaps, we bin counties into 20 bins based on percent votes for GOP candidates, in order to capture differences in the percent votes while preventing an excessive number of empty bins. In terms of statistical methods, we estimate the effects of politics and other variables on migration using multiple ordinary least squares (OLS) regression [25]. However, the migrant networks also exhibit network dependencies that violate the independence assumption used for statistical inference with OLS. (That is, they have node-level (e.g. sender effects, receiver effects, and activity effects) and edge-level covariates (e.g. homophily, heterophily, and mixing matrices) that influence the migration patterns). Thus, we use a network-based permutation testing, specifically, the Quadratic Assignment Procedure (QAP) [26], for the hypothesis testing stage to calculate p-values for the regression coefficients [27].

## Results

### Polarized migration patterns

**Fig 2:**
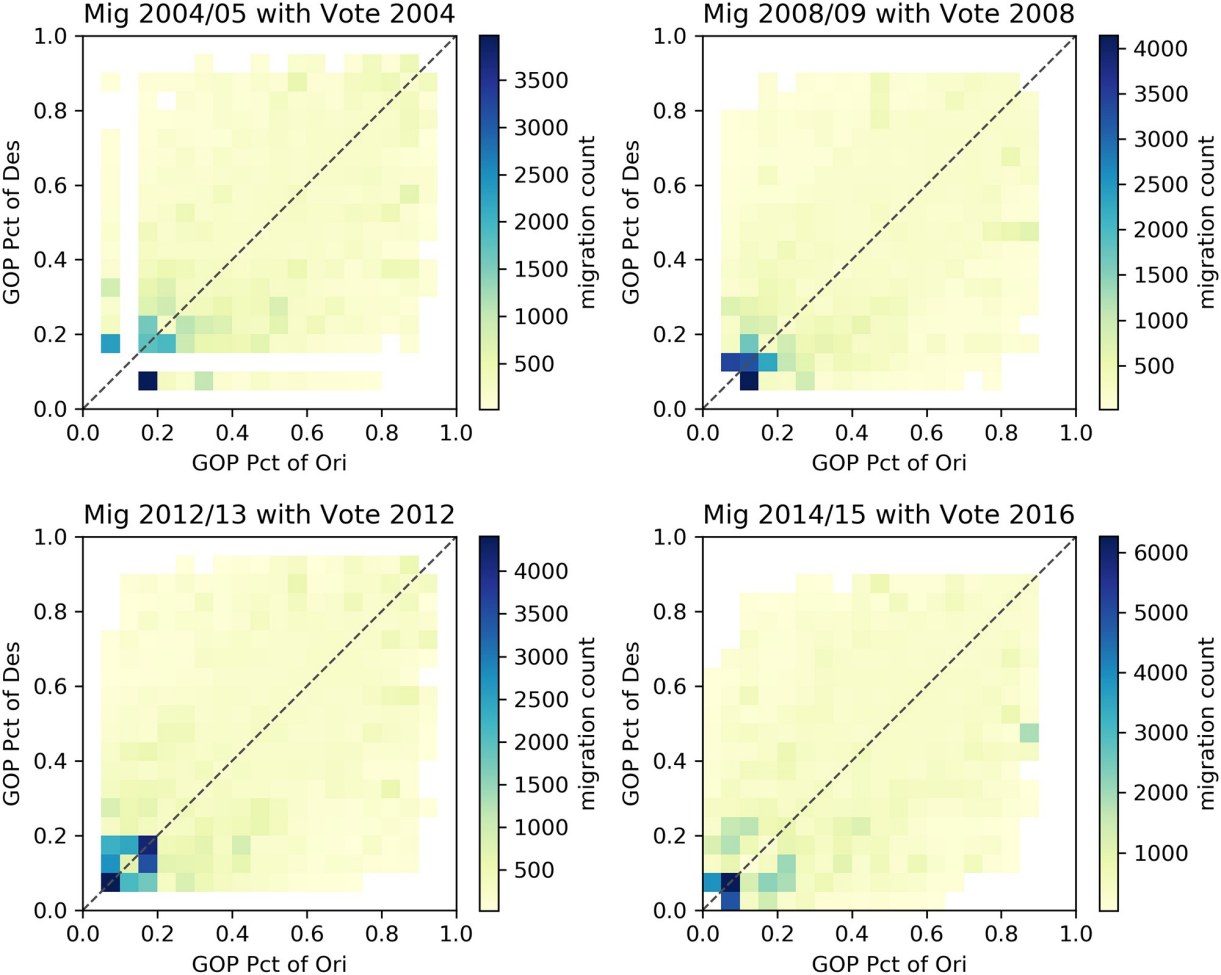
Heapmaps based on raw migration data. The following heatmaps depict the average number of migrants from counties with certain GOP presidential voting rates (x axis) to counties with certain GOP rates (y axis).

**Fig 3:**
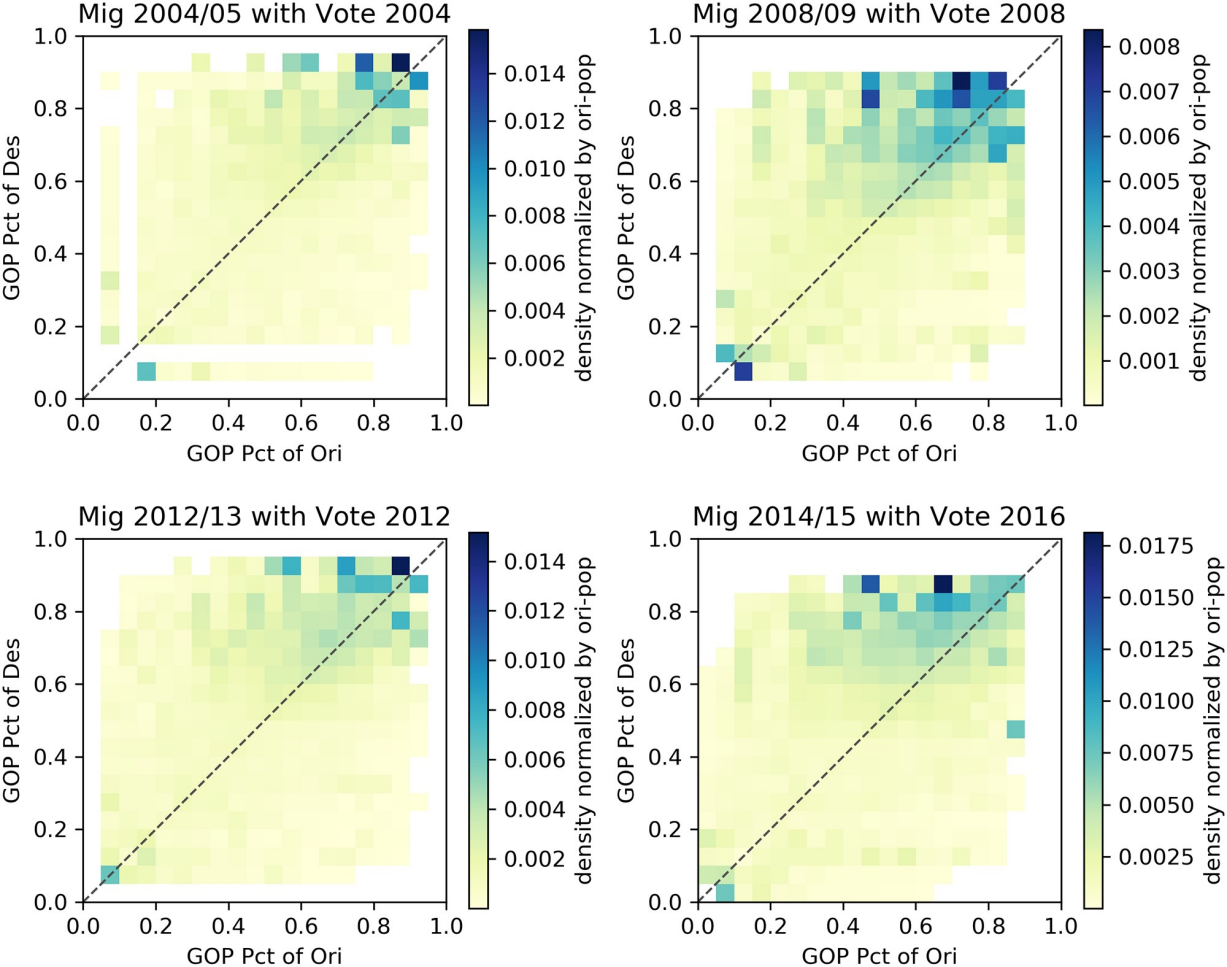
Heatmaps of migrants normalized by population of origin counties. The following heatmaps depict the average number of migrants from counties with certain GOP presidential voting rates (x axis) to counties with certain GOP rates (y axis). Values are normalized by the population of origin counties.

**Fig 4:**
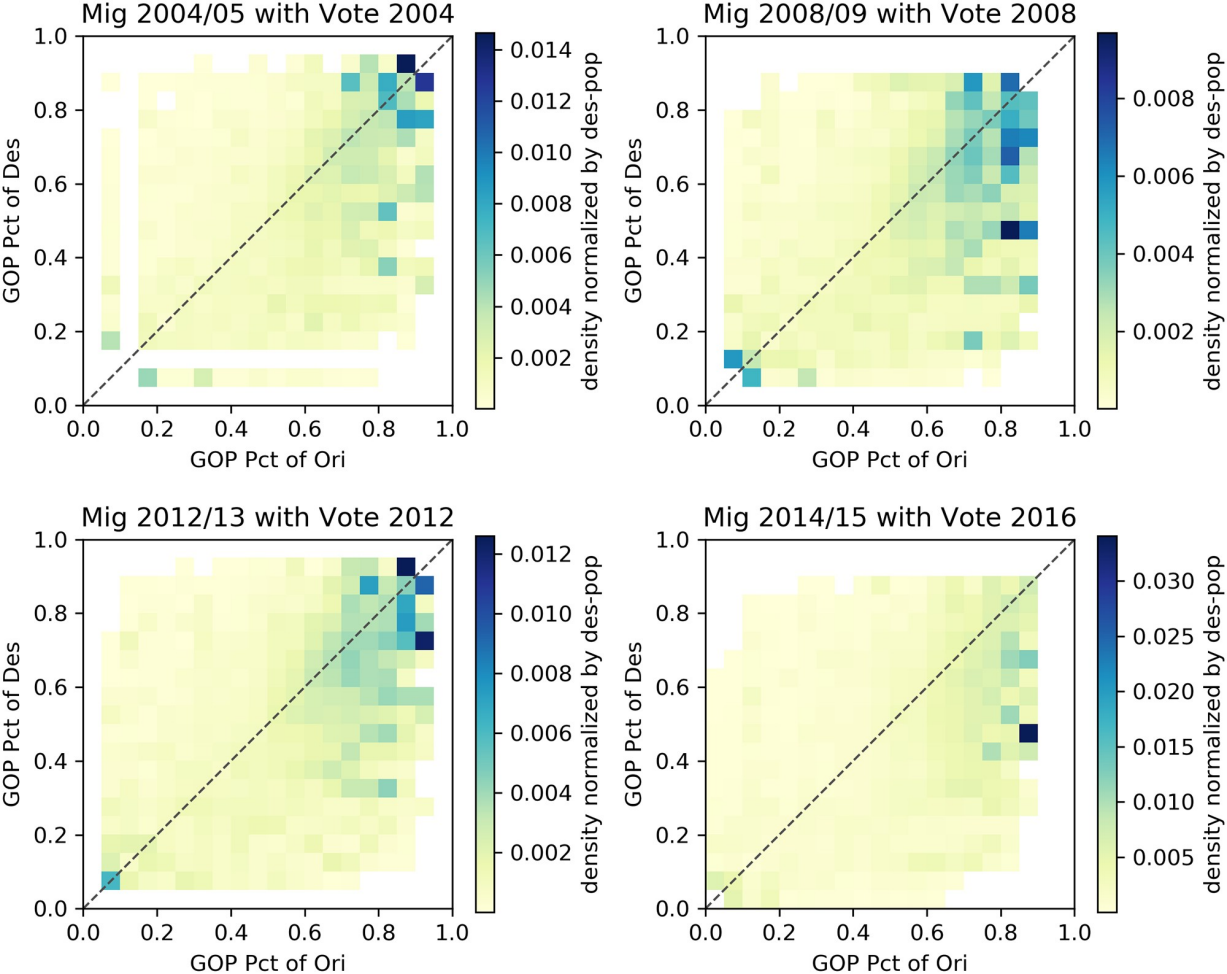
Heatmaps of migrants normalized by population of destination counties. The following heatmaps depict the average number of migrants from counties with certain GOP presidential voting rates (x axis) to counties with certain GOP rates (y axis). Values are normalized by the population of destination counties.

**Fig 5:**
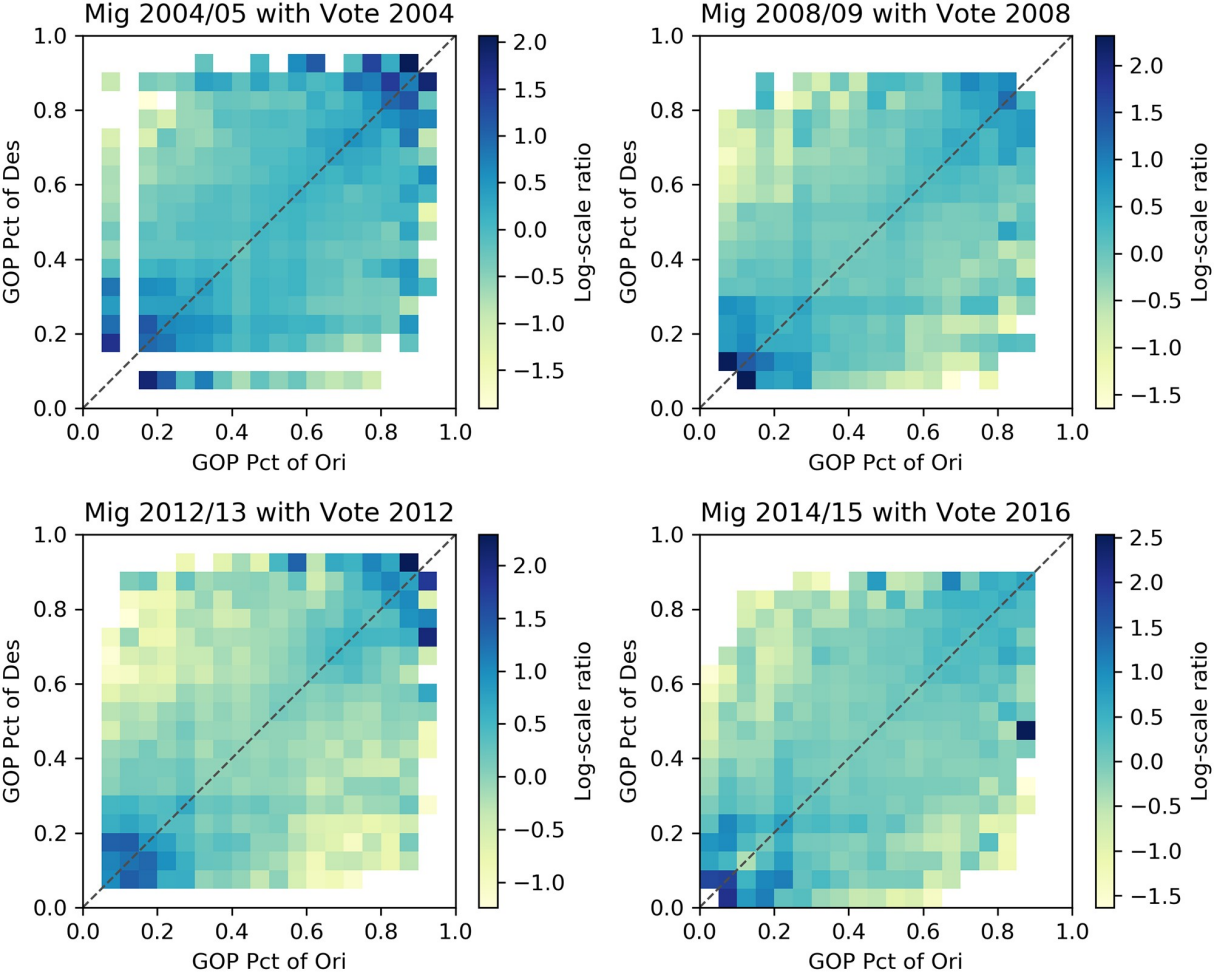
Heatmaps of migrants normalized by the gravity model. The following heatmaps depict the log-scale ratio between the volume of actual migration flows and those estimated using a gravity model.

Figs 2–5 describe heatmaps of migration intensity between counties conditioned on the partisan preferences of the origin and destination counties during the two-year periods of 2004–2005, 2008–2009, 2012–2013, and 2014–2015. The four periods correspond to four presidential elections in the years 2004, 2008, 2012, and 2016. Heatmaps for other years are listed in the S1 Appendix. Each figure depicts the relationship based on a different measure of migration flow intensity. In Fig 2, the intensity is measured by the average number of migrants for each pair of counties, given the percentage of GOP voters in those counties. In Fig 3, the average number of migrants is normalized by origin county population for the given years and in Fig 4, by the destination county. In Fig 5, intensity is described by the log-scale ratio between the volume of actual migration flows and those estimated using a gravity model (See S1 Appendix for more detail).

Though the exact structure of homophily varies slightly based on the particular measure of migration intensity, the heatmaps reveal a consistent form of homophily that departs from a conventional homophily pattern. A conventional homophily pattern would indicate that high migration intensity is observed in regions of the plots with similar party voting proportions (e.g., when the proportion voting Republican was nearly equal in both counties). However, for none of the years, and none of the intensity measures, do we observe notably high intensity among moderate counties. Instead, we observe that flows between similar extreme partisan counties are the most intense, and flows involving moderate counties do not exhibit strong homophily. The implication of this pattern is that those moving from moderate partisan counties are equally likely to move to extreme partisan counties as they are to other moderate counties, but those moving from an extreme partisan county are likely to move to a politically similar extreme county. According to this pattern, extremely partisan counties would operate as magnets—drawing population from moderate counties, and then exchanging with other extreme counties. This could serve as a mechanism that perpetuates greater county-level partisan polarization over time. In the next section, we present statistical analyses in which we adjust for other possible explanations of the identified patterns.

### Regression analysis

The patterns evident in the descriptive heatmaps above suggest homophily in geographic flows originating in extreme partisan counties. However, this finding may be driven by the effects of confounding variables that are not represented in the descriptive analysis. For example, it is well known, and illustrated in Fig 1, that rural areas are more heavily Republican, and densely populated urban areas are more heavily Democratic (see also S1 Appendix). The findings documented in the previous section may therefore, for example, be driven by a tendency for people to move from large cities to other large cities. Furthermore, migration literature posits that multiple socioeconomic factors drive migration patterns—many of which may be correlated with partisan preferences, and thus confound the inferences drawn from our initial bivariate analyses. In this section, we present a statistical analysis designed to adjust for confounding factors, and isolate the relationship between the partisanship of origin/destination counties and migration flows.

We use regression methods to account for other factors that may affect migration flows, and isolate the relationship between county partisanship and migration. The linear regression model we estimate takes the form

$$y_{i,j,t}=\beta_{0,t}+\sum_{k=1}^{K}\beta_{k,t}\times x_{i,j,t}^{\left(k\right)}+\epsilon_{i,j,t},$$

where *y*_*i*,*j*,*t*_ is the migration flow from county *i* to county *j* during time period *t*, *β*_0,*t*_ is an intercept term that controls the overall level of migration at time *t*, $x_{i,j,t}^{\left(k\right)}$ is one of *K* predictor variables used to model *y*_*i*,*j*,*t*_, *β*_*k*,*t*_ is a regression coefficient that determines the effect of $x_{i,j,t}^{\left(k\right)}$ on *y*_*i*,*j*,*t*_, and *ϵ*_*i*,*j*,*t*_ is an error term that reflects the deviation of *y*_*i*,*j*,*t*_ from its expected value. Linear regression is commonly used to decompose the effects of several variables on migration flows [28–31]. The coefficients are interpreted as measures of the effects of the variables on the expected value of *y*_*i*,*j*,*t*_, and the performance, or fit, of the model is assessed based on the percentage of the variance in the *y*_*i*,*j*,*t*_ that can be explained by the independent variables, or adjusted *R*^2^.

The specified regression model contains features that have been found in the past to affect migration in the United States, including whether the origin and destination counties are in the same state, as well as counties’ population, median age [32, 33], median income [28], education (measured by percent of population with a bachelor’s degree) [33], unemployment rate [28], median house price [32], and Haversine distance between county centers. To further control for the overall effects of homophily with respect to industry composition, among the industry effects, we also include the cosine similarity between the industry compositions of two counties, defined at the level of 2-Digit NAICS Codes. The rationale behind testing for industry composition homophily is to capture the phenomenon wherein workers with a certain skill set may be drawn to areas with large employment sectors in their profession.

For political factors, we include a standard specification for homophily (Table 1) that includes GOP support rate of the origin county (‘GOP_send’), GOP support rate of the destination county (‘GOP_receive’), and the absolute difference of GOP support rate between origin and destination counties (‘GOP_diff’). We also include two more metrics that are designed to model the pattern according to which homophily is more intense among more extreme counties. We refer to these two terms as ‘GOP_shared_bias’ and ‘GOP_prod’. We now define these two terms. First, let the GOP bias of a county be *τ*_*i*_ = *GOP*_*i*_ − 0.5, where *GOP*_*i*_ is the proportion in county *i* voting for the GOP candidate. We define the ‘GOP_ shared_bias’ of county *i* and *j* to be max(0, min(*τ*_*i*_ ⋅ sign(*τ*_*j*_), *τ*_*j*_ × sign(*τ*_*i*_))). For example, if two counties have partisan biases of 0.1 and 0.3, respectively, GOP_shared_bias would be 0.1; if two counties have partisan bias of 0.1 and -0.1, the GOP_ shared_bias would be 0. In model 5, we include the shared bias GOP_ shared_bias and the product GOP_prod = GOP_shared_bias × GOP_diff. The GOP_shared_bias measures the degree to which two counties are extreme in the same direction. The GOP_prod conditions the homophily effect on the shared extremeness of the two counties. The vote-related variables are, due to their relationships with each other, difficult to interpret in isolation. We use visualizations of combined effects to understand the patterns according to which voting is relate. Note, our model specification can be seen as an extension of the model in Charyyev and Gunes (2019) [14], which includes the effects of county-level presidential voting on total in- and out- migration, but does not model the effects of partisan homophily along with other variables. In Tables 2 and 3, we summarize the factors included in each estimated model.

**Table 1:** Explanation of variable terms used in the models.

Variable term	Description
GOP_send	Fraction of people who voted for a Republican presidential candidate in the origin county.
GOP_receive	Fraction of people who voted for a Republican presidential candidate in the destination county.
GOP_diff	Absolute value of difference in fraction of people who voted for a Republican presidential candidate in origin vs. destination county.
GOP_shared_bias	Shared deviation in GOP_send and GOP_receive from 0.5 (e.g., if origin is 0.6, and destination is 0.7, shared bias is 0.1; if origin is 0.3, and destination is 0.2, shared bias is 0.2; if origin is 0.6, and destination is 0.3, shared bias is 0).
GOP_prod	GOP_diff × GOP_shared_bias—testing whether homophily is stronger in pairs of counties that are more politically extreme (e.g., is homophily stronger between a 0.9 county and 0.8 county than between a 0.45 county and a 0.55 county).

**Table 2:** Variables in the two groups of models.

Variables	Model Group A	Model Group B
Geographical Effects	Same State	Same State
	Inverse Distance	Log-scale Distance
	Population (send, rec, dist, prod)	Log-scale Population (send, rec, dist)
Fixed County Effects	Median Income (send, rec, dist)	Log-scale Median Income (send, rec, dist)
	Median Housing Value (send, rec, dist)	Log-scale Median Housing Value (send, rec, dist)
	Median Age (send, rec, dist)	Median Age (send, rec, dist)
	Pct Bachelor Degree (send, rec, dist)	Pct Bachelor Degree (send, rec, dist)
	Unemployment Rate (send, rec, dist)	Unemployment Rate (send, rec, dist)
Industry Effects	NAICS 2-Digit Codes (cosine similarity)	NAICS 2-Digit Codes (cosine similarity)

**Table 3:** Summary of independent variables included in each model.

Variables	Model 1	Model 2	Model 3	Model 4	Model 5
Geographical Effects	Yes	Yes	Yes	Yes	Yes
Fixed County Effects		Yes	Yes	Yes	Yes
Industry Effects			Yes	Yes	Yes
GOP_send				Yes	Yes
GOP_receive				Yes	Yes
GOP_diff				Yes	Yes
GOP_shared_bias					Yes
GOP_prod					Yes

We use two groups of models in order to test the robustness of the polarization patterns. Models in group A predict log-scale migration flows, while all the other variables are in the original format. They also include the product of population in origin/destination counties and inverse distance as independent variables. Models in group B are based on the log-scale format gravity model, since the gravity model is often used in the log format [34, 35] i.e.

$$log\left(flow_{ij}\right)=\alpha_{1}log\left(P_{i}\right)+\alpha_{2}log\left(P_{j}\right)+\beta log\left(d_{ij}\right)+\gamma ,$$

where *P*_*i*_ represents the population for county *i*, and *d*_*ij*_ represent the geographical distance between the county *i* and county *j*. We thus use the log format of population of both origin and destination counties in the model, and also log scale for median income and median housing value. Other variables, including GOP supporting rate, median age, percent of the population with bachelor’s degrees, unemployment rate, and employees in various sectors are represented using the original scale (Table 2). Since network data, such as migration flows, are characterized by complex dependence [17] (e.g., the flow from *i* to *j* may depend on the flow from *j* to *i*, or there may be a tendency for flows to cluster in triads), we use a hypothesis testing method, Quadratic assignment procedure (QAP), that is designed for hypothesis-testing for regression with data characterized by network dependence and is robust to network dependence [36]. The P-values we report are based on 500 iterations of QAP permutations.

In Table 4 we present the adjusted *R*^2^ values for each model estimated. In both model groups, the full model, which includes the complete list of control variables and vote preference effects on migration, provides the best fit to the data. This serves as evidence that we can interpret the relationships between political variables and migration flows as contributing to the models’ explanatory power, both across model types and over years. However, it is important to note that the variables related to politics increase the adjusted *R*^2^ by a modest 1–3%, indicating both that politics is not the dominant feature driving migration, and the importance of using the regression framework to adjust for other explanatory factors.

**Table 4:** Adjusted *R*^2^ values of different models.

Model Group	Year	Model 1	Model 2	Model 3	Model 4	Model 5
A	02-03	0.4268	0.4319	0.4330	0.4338	0.4341
	04-05	0.4254	0.4305	0.4316	0.4325	0.4328
	06-07	0.4376	0.4429	0.4439	0.4440	0.4443
	08-09	0.3610	0.3679	0.3688	0.3690	0.3693
	10-11	0.4441	0.4479	0.4491	0.4492	0.4496
	12-13	0.4389	0.4426	0.4438	0.4439	0.4443
	14-15	0.3851	0.3888	0.3897	0.3899	0.3904
B	02-03	0.2266	0.2370	0.2386	0.2393	0.2414
	04-05	0.2317	0.2419	0.2436	0.2442	0.2462
	06-07	0.2348	0.2462	0.2480	0.2486	0.2510
	08-09	0.2297	0.2407	0.2425	0.2431	0.2456
	10-11	0.2356	0.2439	0.2458	0.2467	0.2499
	12-13	0.2226	0.2305	0.2323	0.2331	0.2361
	14-15	0.1594	0.1662	0.1674	0.1694	0.1762

**Fig 6:**
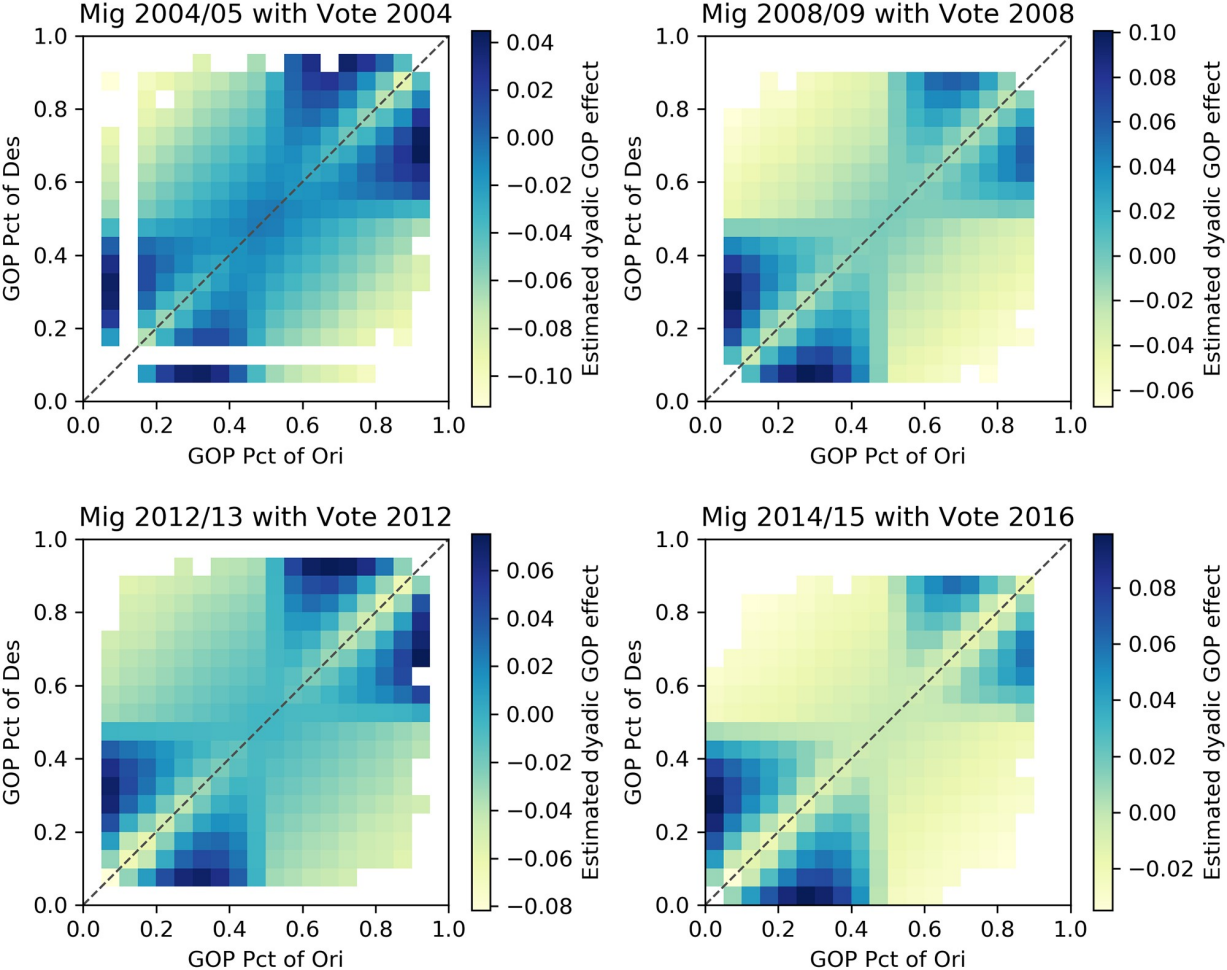
Dyadic effects for model group A. The integrated dyadic terms’ effects based on GOP_diff, GOP_shared_bias, and GOP_prod between counties with certain proportion votes supporting the GOP candidate for model group A.

**Fig 7:**
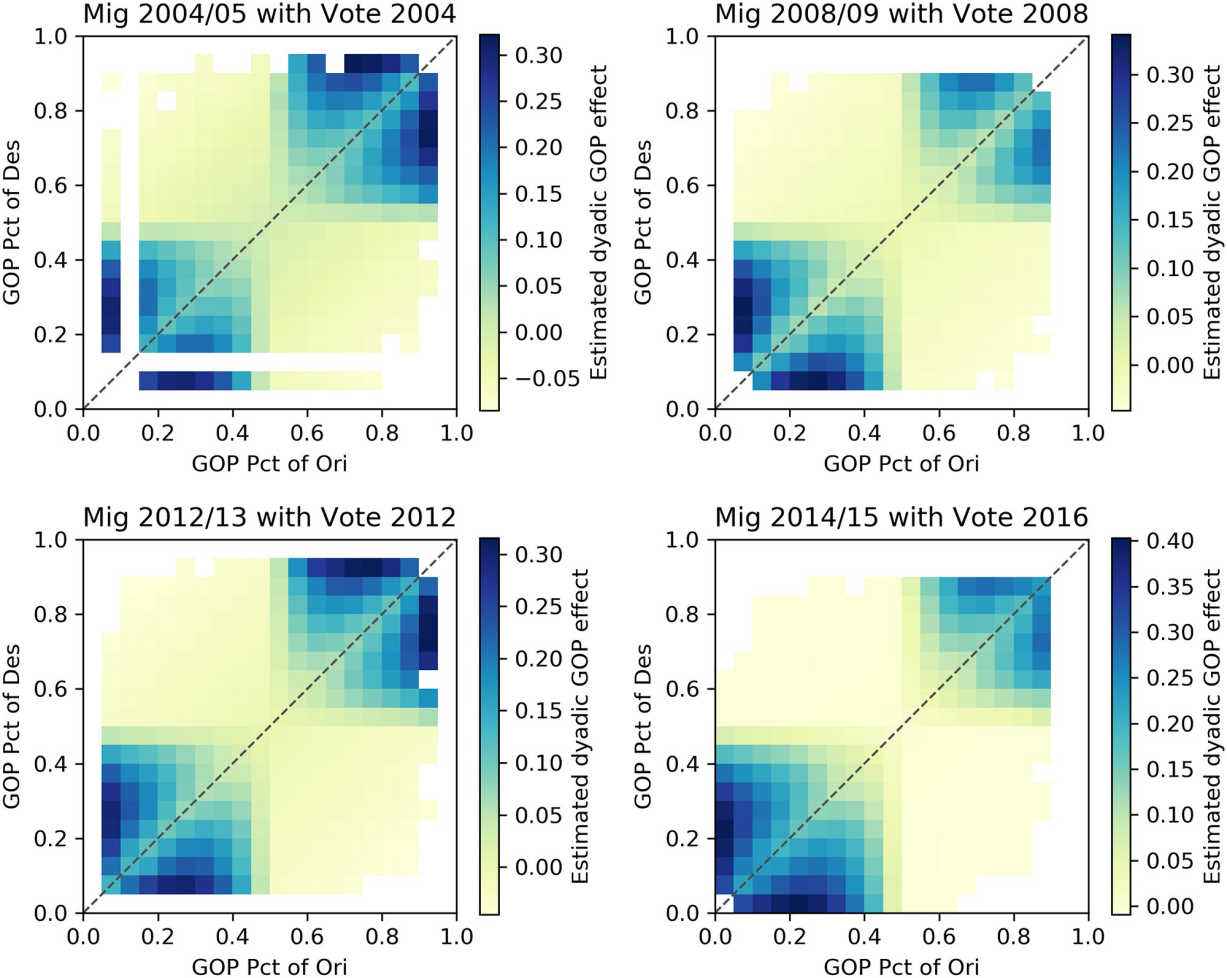
Dyadic effects for model group B. The integrated dyadic terms’ effects based on GOP_diff, GOP_shared_bias, and GOP_prod between counties with certain proportion votes supporting the GOP candidate for model group B.

The results of coefficients for political factors are presented in Table 5. In most models in both groups, the coefficient value of GOP_diff is negative, indicating that migration tends to connect counties with similar voting patterns. Moreover, by calculating the dyadic effects based on GOP_diff, GOP_shared_bias, and GOP_prod, Figs 6 and 7 indicate that the relationship between county migration flows and partisan composition is one of homophily with respect to partisan composition. That is, adjusting for other factors that might affect migration flows, we find that flows are higher between counties with similar partisan compositions, and that this effect is particularly strong for counties with relatively extreme partisan compositions. In addition to the statistical significance of the partisan composition variables, the results we report also show that the model fit, assessed using adjusted R^2^, improves in each year as a result of adding the partisan composition variables (Table 4).

**Table 5:** Result summary of model groups A and B.

Type, Model	Year	GOP diff	GOP send	GOP receive	GOP shared bias	GOP prod
A, 4	02-03	-0.094***	0.095***	0.140***		
	04-05	-0.082***	0.096***	0.164***		
	06-07	-0.032⋅	0.004	0.059**		
	08-09	-0.082***	0.005	0.031		
	10-11	-0.044*	-0.002	0.030		
	12-13	-0.032⋅	0.001	0.042*		
	14-15	-0.033**	-0.047***	-0.028		
A, 5	02-03	-0.160***	0.095**	0.139***	-0.260***	2.968***
	04-05	-0.147***	0.095**	0.163***	-0.258***	2.991***
	06-07	-0.070*	0.002	0.056*	-0.243***	3.310***
	08-09	-0.096***	-0.013	0.014	-0.129*	3.183***
	10-11	-0.084**	-0.018	0.014	-0.223***	3.293***
	12-13	-0.074*	-0.009	0.032	-0.229***	2.986***
	14-15	-0.051*	-0.076***	-0.056**	-0.101*	2.547***
B, 4	02-03	-0.175***	-0.044	0.020		
	04-05	-0.165***	-0.038	0.044		
	06-07	-0.134***	-0.102***	-0.040		
	08-09	-0.132***	-0.088**	-0.059⋅		
	10-11	-0.151***	-0.101***	-0.068*		
	12-13	-0.139***	-0.097***	-0.052⋅		
	14-15	-0.161***	-0.161***	-0.137***		
B, 5	02-03	-0.122*	-0.247***	-0.184***	0.276*	6.280***
	04-05	-0.111*	-0.245***	-0.163***	0.277*	6.427***
	06-07	-0.068	-0.200***	-0.138***	0.188⋅	6.663***
	08-09	-0.067	-0.186***	-0.157***	0.180⋅	6.717***
	10-11	-0.069	-0.250***	-0.217***	0.284**	6.046***
	12-13	-0.061	-0.238***	-0.192***	0.274**	5.644***
	14-15	-0.014	-0.365***	-0.341***	0.524***	5.178***

P-values:

*** <0.001,

** <0.01,

* <0.05,

⋅ <0.1

## Discussion

In the United States, the representative democratic system is largely based on the geographic distribution of partisan preferences. On the national political stage, representation in both the Senate and the Electoral College are allocated with a bias that favors residents of low-population states. Geographic disparities in partisan preferences, which will persist under the patterns of partisan migration we have documented, exacerbate representational inequality.

We present a comprehensive analysis of the ways in which migration flows between counties are conditioned by the partisan composition of counties. The consistent pattern, which is evident in both bivariate heatmaps and multiple regression models, shows that the migration network is characterized by a lack of homophily among moderate counties, and strong homophily among extreme counties. This pattern, which shows no sign of reversing, could serve as a mechanism through which partisan polarization of counties is exacerbated. Our findings suggest that those with politically extreme preferences are more likely self-select into ideologically homogeneous locales—a result that deserves attention in future research on individual mobility patterns. Our results fit with and build upon a growing body of literature that highlights the significance of political factors in shaping individual attitudes and aggregate patterns related to migration.

In addition, we find that explanatory variables such as the gravity model, and magnetism between places with similar pairs of industrial compositions, help explain higher rates of migration. The gravity model variable emphasizes the convenience of migrating to a nearby location. The self-selection of industry similarities between origins and destinations emphasizes the lack of importance of geographic distance for those who “live life in the network” [37] of similar employment divisions.

## Supporting information

S1 Appendix More information on data, heatmaps, and the QAP method. (PDF)
