# Supplementary material for: Migration and political polarization in the U.S.: An analysis of the county-level migration network
Source: PLoS One. 2019 Nov 22;14(11):e0225405. doi: 10.1371/journal.pone.0225405 (PMC6874318; doi:10.1371/journal.pone.0225405)
Supplement: S1 Appendix — (PDF) [file pone.0225405.s001.pdf]

Supplementary Information: Migration and Political Polarization in the U.S.: An Analysis of the County-level Migration Network

Data

We used counties of population 20,000 or more, as recorded in the 2010 Decennial Census. Since distance is an important factor influencing migration behavior, we removed all the migration flows to and from counties in Alaska and Hawaii. We also used the presidential voting data, employment data and census data that are closest to the year of migrations to construct the regression models (Table S2, S3). The county-level presidential election returns data [1] did not have values for total votes in 2004 for counties in Oklahoma, so we used the sum of voters of GOP and DEM when calculating the voting rate for GOP.

NAICS Data

Employment data by industry was retrieved from the U.S. Department of Labor's Bureau of Labor Statistics. The data collection is called Quarterly Census of Employment and Wages, and the data used is from the North American Industry Classification System (NAICS), available online at: https://www.bls.gov/cew/downloadable-data-files.htm. The dataset used here is entitled "Single File CSVs on Annual Averages", and numbers represent number of employees in a particular sector. The data is aggregated to the sector level (aggregation level 76, representing 2-digit codes). The data include annual sums for years 2013 and 2016 and are spatially-aggregated to the county level.

Code descriptions (Table S1) are sourced from the U.S. Executive Office of the President, Office of Management and Budget (2017), retrieved from https://www.census.gov/eos/www/naics/2017NAICS/2017_NAICS_Manual.pdf.

Heatmaps for Migration Flows

We used heatmaps to show the density of migrant flows between county pairs with different GOP voting percentages. In the four presidential elections, most migrants flow between relatively neutral

Table S1: North American Industry Classification System (NAICS) Codes

Two-Digit Code	Industry Description
11	Agriculture, Forestry, Fishing and Hunting
21	Mining, Quarrying, and Oil and Gas Extraction
22	Utilities
23	Construction
31-33	Manufacturing
42	Wholesale Trade
44-45	Retail Trade
48-49	Transportation and Warehousing
51	Information
52	Finance and Insurance
53	Real Estate and Rental and Leasing
54	Professional, Scientific, and Technical Services
55	Management of Companies and Enterprises
56	Administrative and Support and Waste Management and Remediation Services
61	Educational Services
62	Health Care and Social Assistance
71	Arts, Entertainment, and Recreation
72	Accommodation and Food Services
81	Other Services (except Public Administration)
92	Public Administration

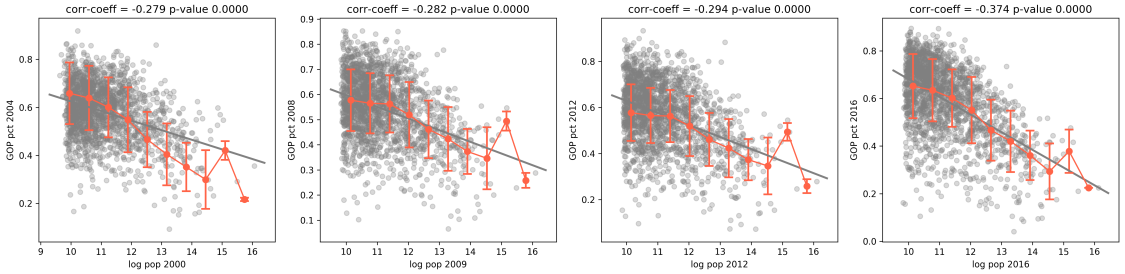

Figure S1: Low-population counties tend to vote for GOP candidates throughout the years.

Table S2: Data Summary

Data Type	Available Years	Data Type	Available Years
IRS Migration Data	2002 - 2015	Presidential Election Voting Data	2004, 2008, 2012, 2016
Population	2000, 2009, 2012, 2016	Median Household Income	2000, 2009, 2012, 2016
Median Age	2000, 2009, 2012, 2016	Median Value of Owner-Occupied Housing Units	2000, 2009, 2012, 2016
Unemployment Rate	2000, 2009, 2012, 2016	Percent of Population with Bachelor's Degree	2000, 2009, 2012, 2016
NAICS 2-Digit Codes	2013, 2016		

counties (Figure S2 and Figure S3), as counties with relatively neutral voting patterns are common in the U.S. system.

In the main document, we presented a number of heatmaps of raw flows, and flows normalized by county origin population, county destination population. Each revealed consistent patterns of homophily throughout the years. In the main document these heatmaps are shown for four time periods, and here, we extend this to all seven time periods (Figure S4, Figure S5, and Figure S6), to illustrate more temporal detail.

Finally, we used the gravity model to estimate migrant flow ($flow(i,j)$) and compare the difference between actual and estimated migration flows. The gravity model is mathematically estimated by:

$$flow(i,j) = k\frac{P_1^{\alpha_1} P_2^{\alpha_2}}{d_{ij}^{\beta}}$$

and we calibrated coefficients ($k, \alpha_1, \alpha_2, \beta$) using actual migration flows. Here, county pairs with similar rates of partisan voting are more strongly connected (Figure S7).

Regression with QAP

We used Quadratic Assignment Procedure (QAP) [3] for the hypothesis-testing stage. QAP is suitable for testing significance when independent variables are network adjacency matrices, as evidenced by prior political network related studies [2]. As introduced in the main paper, we calculated the dyadic effects resulting from political polarization based on GOP_diff, GOP_shared_bias, and GOP_prod. In both groups of models (group A and B), migration data throughout the years demonstrated strong tendencies to connect places with similar voting patterns (Figure S8 and Figure S9). Flows were higher between counties with similar partisan compositions, and this effect was particularly strong for counties with relatively extreme partisan compositions.

Table S3: Data combination for regression

Model Sequence	Migration Data	Census Data	NAICS Data	Voting Data
1	2002 & 2003	2000	2013	2004
2	2004 & 2005	2000	2013	2004
3	2006 & 2007	2009	2013	2008
4	2008 & 2009	2009	2013	2008
5	2010 & 2011	2012	2013	2012
6	2012 & 2013	2012	2013	2012
7	2014 & 2015	2016	2016	2016

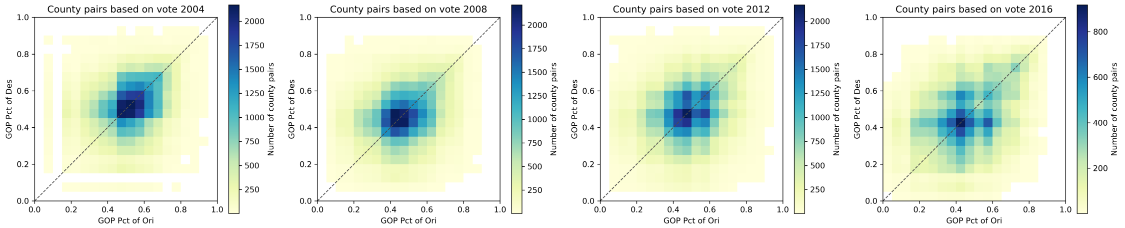

Figure S2: Density of number of county pairs based on specific GOP voting rates in different years.

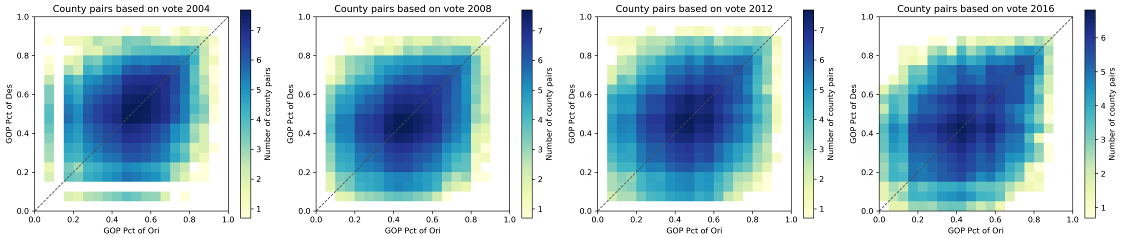

Figure S3: Density of number of county pairs based on specific GOP voting rates in different years using log scale.

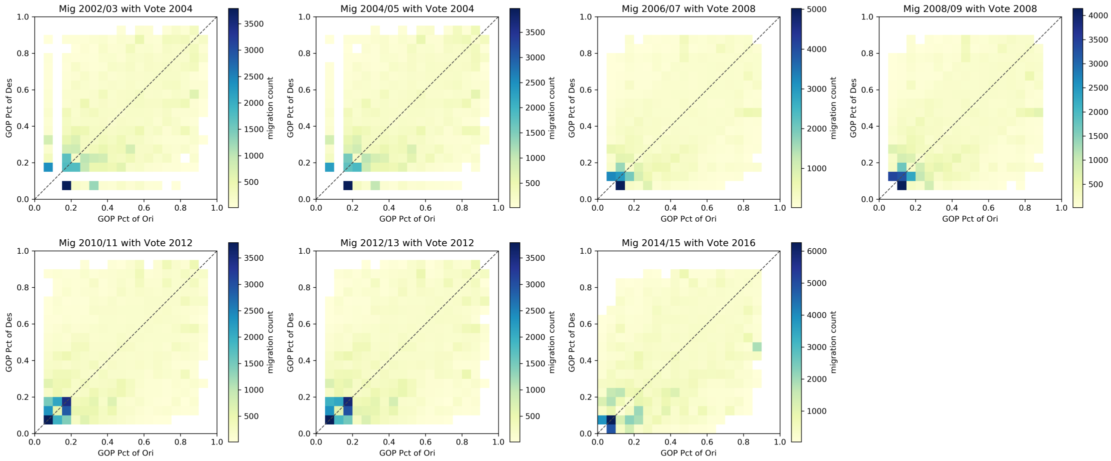

Figure S4: Density of raw migration flows between county pairs with specific GOP voting rates.

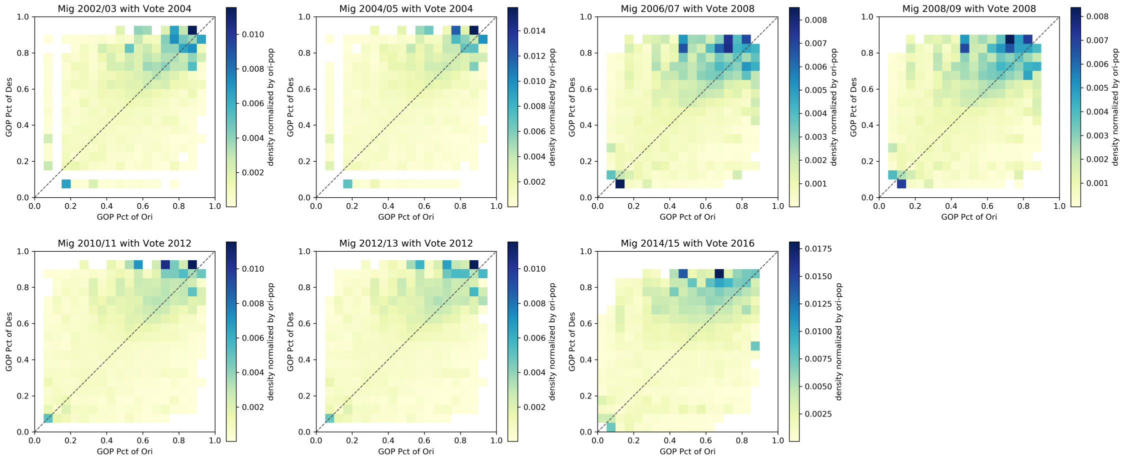

Figure S5: Density of migration flows normalized by origin county population.

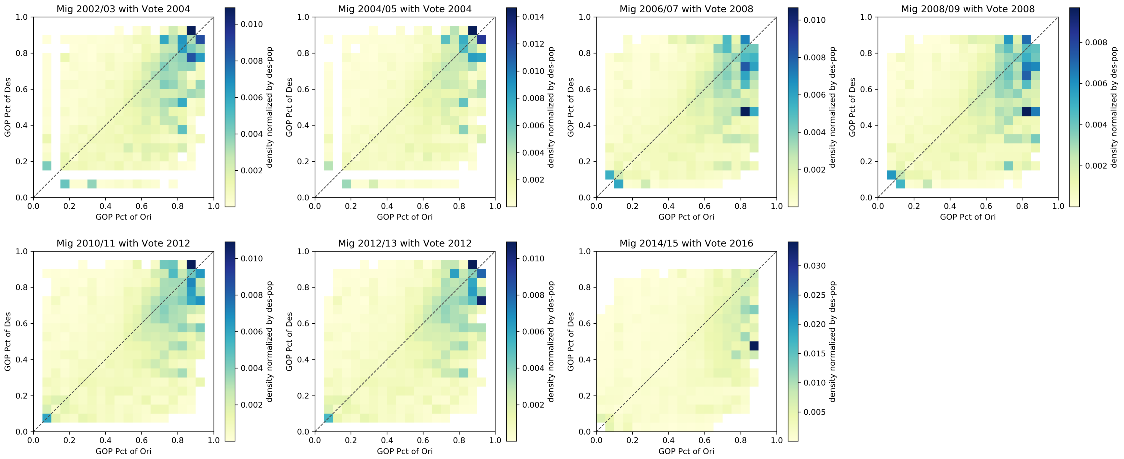

Figure S6: Density of migration flows normalized by destination county population.

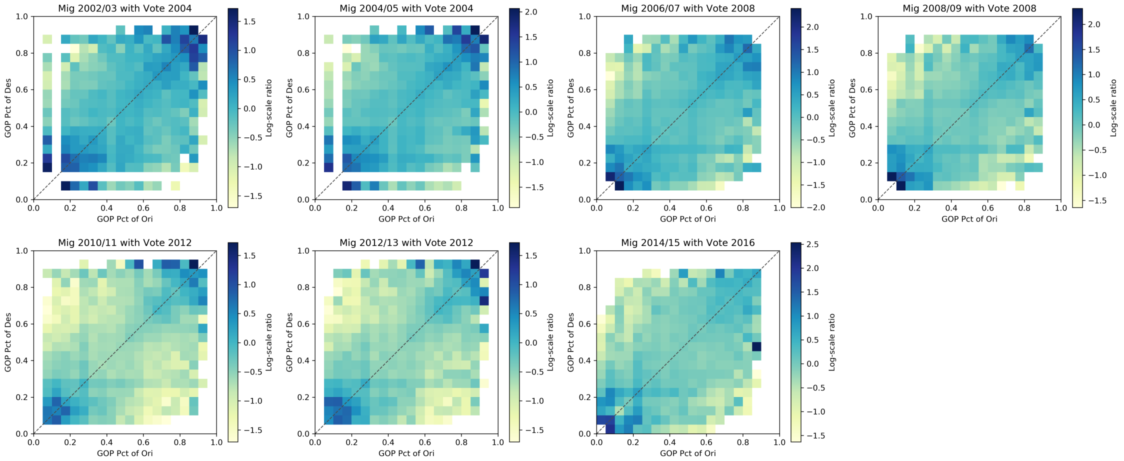

Figure S7: Log-scale ratios between the volume of actual migration flows and estimated migration flows normalized by gravity model estimates.

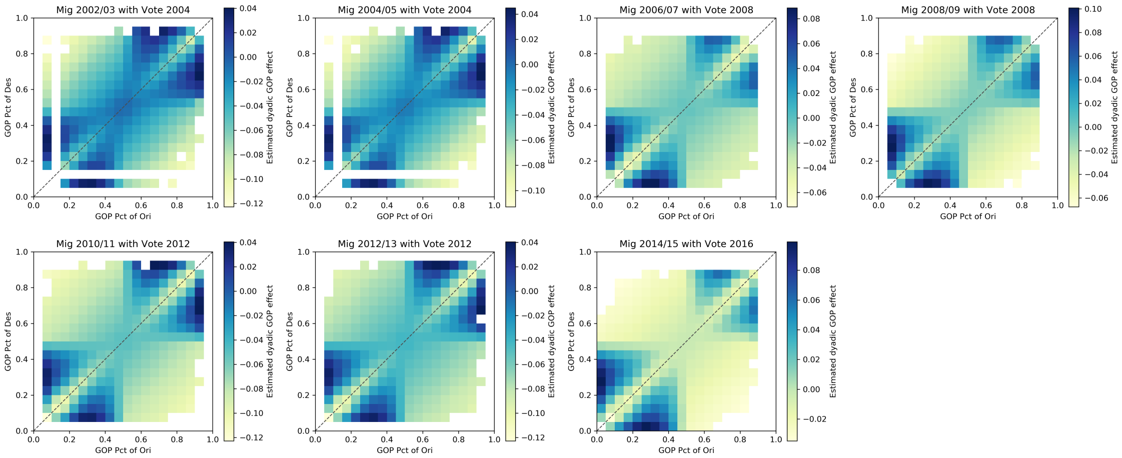

Figure S8: The integrated dyadic terms' effects based on GOP_diff, GOP_shared_bias, and GOP_prod between counties with certain GOP voting rates for model group A.

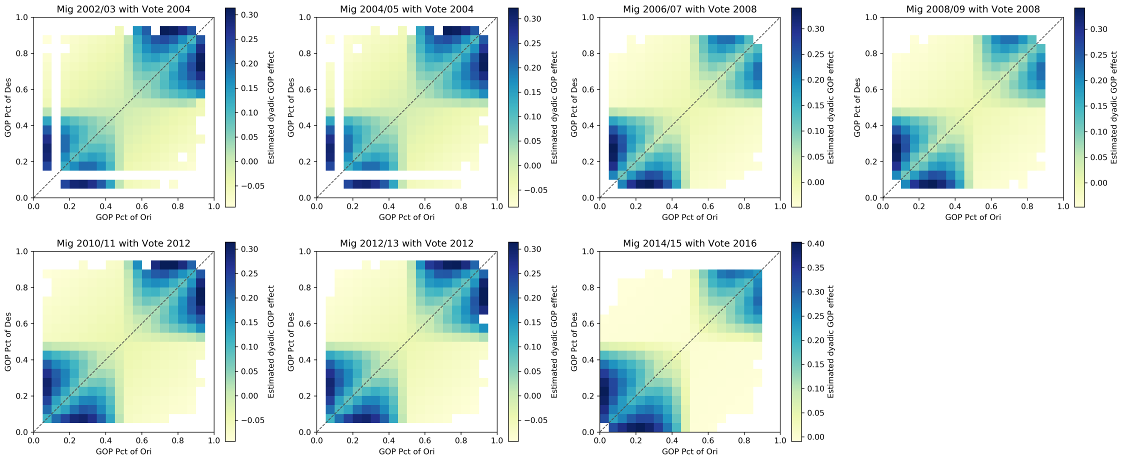

Figure S9: The integrated dyadic terms' effects based on GOP_diff, GOP_shared_bias, and GOP_prod between counties with certain GOP voting rates for model group B.

References

[1] MIT Election Data and Science Lab. County Presidential Election Returns 2000-2016, 2018.

[2] Bruce A Desmarais and Skyler J Cranmer. Statistical inference in political networks research. In Jennifer Nicoll Victor, Alexander H. Montgomery, and Mark Lubell, editors, *The Oxford Handbook of Political Networks*, page 203. Oxford University Press, Oxford, UK, 2017.

[3] David Krackardt. QAP partialling as a test of spuriousness. *Social Networks*, 9(2):171–186, 1987.
